# The effects of six months of exercise on single‐ and dual‐task gait and functional mobility relative to usual care alone among people living with dementia: A pilot randomized controlled trial

**DOI:** 10.1002/alz.087083

**Published:** 2025-01-09

**Authors:** Ryan L Langston, Yanbin Dong, Haidong Zhu, Andre Soares, Charmi Patel, Colleen Hergott, Jennifer L Waller, Lufei Young, Dawnchelle Robinson‐Johnson, Ryan M Carrick, Richard Sams, Mark Hamrick, Deborah A Jehu

**Affiliations:** ^1^ Augusta University, Augusta, GA USA; ^2^ University of North Carolina, Charlotte, NC USA; ^3^ Claiborne Assisted Living Facility, Augusta, GA USA; ^4^ Georgia War Veterans Nursing Home, Augusta, GA USA

## Abstract

**Background:**

Exercise may improve dual‐tasking and mobility impairments among people living with dementia (PWD), but more evidence is needed. The purpose of this pilot randomized controlled trial (RCT) was to determine the effect of six months of exercise on single‐ and dual‐task mobility compared to usual care alone in PWD.

**Method:**

This assessor‐blinded RCT (1:1) included *n* = 21 PWD in the usual care and n = 21 PWD in the exercise group at two residential care facilities (Age = 82 years, 35% female, Montreal Cognitive Assessment (MoCA) = 10.2±5.9; NCT05488951). The physical therapist‐led adapted Otago Exercise Program involved 30 minutes of lower body strength and balance exercises and 30 minutes walking 3x/week for 6 months in groups of 5‐7. At baseline and 6 months, participants completed two trials of single‐ (walk 4m) and dual‐task gait (walk 4m while naming words), and single‐task timed‐up‐and‐go (TUG), and dual‐task TUG with a category task using APDM inertial sensors. We measured double limb support (%), gait speed (m/s), and stride length (m) for gait, and duration (s), turns angle (°), and turn velocity (m/s) for the TUG. Intent‐to‐treat (ITT) and per protocol (all usual care and exercisers with ≥2/3 adherence) analyses were performed. We controlled for age, race, sex, and the MoCA.

**Result:**

In the ITT analysis, exercise provoked faster dual‐task gait speed (+0.03 m/s, p = 0.006), increased single‐task TUG turn velocity (+24.01 m/s, p = 0.001), decreased dual‐task TUG duration (‐11.54 s, p = 0.01), and increased dual‐task TUG turn velocity (+18.96 m/s, p<0.001). In the per Protocol analysis, both the exercise (‐22.04%, p = 0.007, n = 9) and usual care groups (‐18.90%, p<0.001, n = 21) decreased double limb support during single‐task gait. Additionally, only the exercise group increased stride length during dual‐task gait (+0.24 m, p = 0.007) and increased turn velocity during the single‐ (+28.27 m/s, p = 0.002) and dual‐task TUG (+17.75 m/s, p = 0.002).

**Conclusion:**

Six months of exercise improved both single‐ and dual‐task mobility in PWD compared to usual care. Similar findings in both ITT and per protocol analyses indicate that exercising in any amount can improve dual‐task mobility in PWD. Our findings may inform therapeutic interventions for improving dual‐task mobility in PWD in residential care facilities.

